# The interplay between emerging human coronavirus infections and autophagy

**DOI:** 10.1080/22221751.2021.1872353

**Published:** 2021-02-03

**Authors:** Zhenyu Zhao, Kefeng Lu, Binli Mao, Shi Liu, Mirko Trilling, Ailong Huang, Mengji Lu, Yong Lin

**Affiliations:** a Key Laboratory of Molecular Biology of Infectious Diseases (Chinese Ministry of Education), Department of Infectious Diseases, The Second Affiliated Hospital, Institute for Viral Hepatitis, Chongqing Medical University, Chongqing, People’s Republic of China; b Department of Neurosurgery, State Key Laboratory of Biotherapy, West China Hospital, Sichuan University, Chengdu, People’s Republic of China; c State Key Laboratory of Virology, College of Life Sciences, Wuhan University, Wuhan, People’s Republic of China; d Institute for Virology, University Hospital Essen, University of Duisburg-Essen, Essen, Germany

**Keywords:** Autophagy, coronavirus, MERS-CoV, MHV, SARS-CoV, SARS-CoV-2

## Abstract

Following outbreaks of severe acute respiratory syndrome coronavirus (SARS-CoV) and the Middle East respiratory syndrome coronavirus (MERS-CoV) in 2002 and 2012, respectively, the severe acute respiratory syndrome coronavirus 2 (SARS-CoV-2) is the third highly pathogenic emerging human coronavirus (hCoV). SARS-CoV-2 is currently causing the global coronavirus disease 2019 (COVID-19) pandemic. CoV infections in target cells may stimulate the formation of numerous double-membrane autophagosomes and induce autophagy. Several studies provided evidence that hCoV infections are closely related to various cellular aspects associated with autophagy. Autophagy may even promote hCoV infection and replication. However, so far it is unclear how hCoV infections induce autophagy and whether the autophagic machinery is necessary for viral propagation. Here, we summarize the most recent advances concerning the mutual interplay between the autophagic machinery and the three emerging hCoVs, SARS-CoV, MERS-CoV, and SARS-CoV-2 and the model system mouse hepatitis virus. We also discuss the applicability of approved and well-tolerated drugs targeting autophagy as a potential treatment against COVID-19.

## Abbreviations

ACE2: Angiotensin-converting enzyme 2
AKT: protein kinase B
AMPK: AMP-protein activated kinase
ATG: Autophagy-related gene
BECN1: Beclin 1
CQ: Chloroquine
DMV: Double-membrane vesicle
ER: Endoplasmic reticulum
HCQ: Hydroxychloroquine
LC3: Light chain 3
MERS: Middle East respiratory syndrome
MHV: Mouse hepatitis virus
NSP: Non-structural protein
ORF: Open reading frame
RTC: Replication-transcription complex
SARS: Severe acute respiratory syndrome
SKP2: S phase kinase-associated protein 2.

## Introduction

In December 2002, a zoonotic coronavirus (CoV), originating from horseshoe bats, first appeared in China, and was rapidly disseminated, leading to over 8000 confirmed cases at the end of the epidemic [1]. In reference to the clinical disease manifestation, the virus was termed severe acute respiratory syndrome coronavirus (SARS-CoV). A decade later, the Middle East respiratory syndrome coronavirus (MERS-CoV) emerged in Saudi Arabia, causing a SARS-like respiratory disease [2]. Similar to SARS-CoV, MERS-CoV originated from bats; however, in this case, dromedary camels served as intermediate hosts transmitting the virus to humans [3]. Currently, no approved specific antiviral drugs or vaccines are available for the treatment or prophylaxis of SARS-CoV and MERS-CoV infections [4]. Unfortunately, in December 2019, another novel CoV, causing coronavirus disease 2019 (COVID-19) and named as severe acute respiratory syndrome coronavirus 2 (SARS-CoV-2), emerged and spread widely in Wuhan, Hubei province, China [5]. SARS-CoV-2 has a dramatic impact on the health and life expectancy of humankind, impairs the global economy, and endangers socio-economic stability. Scientists and physicians around the world strive to establish efficient diagnostic methods, proper mitigation procedures, antiviral drugs, and prophylactic vaccines. Accumulating evidence suggests that CoV infections affect multiple steps of autophagy, and, *vice versa,* autophagy may also play a crucial role in the viral lifecycle [6–8]. However, the exact mechanisms underlying these mutual interactions between the three emerging human coronavirus (hCoVs) and autophagy are the subject of current research. This review summarizes the latest progress on the interplay between the three emerging hCoVs as well as the model system mouse hepatitis virus (MHV) and autophagy.

## The coronavirus genome and process of virus replication

CoVs are a class of viruses harbouring a positive-sense, single-stranded RNA genome. With a genome size of 27 to 36 kb, CoVs have the largest known viral RNA genomes among viruses infecting mammals [9]. *Coronaviridae* infect birds and mammals, including humans [10]. Most diseases caused by hCoVs predominantly affect the respiratory, intestinal, and nervous systems. In severe cases, infections can be life-threatening, such as in the case of SARS-CoV, MERS-CoV, and SARS-CoV-2 [11]. SARS-CoV and SARS-CoV-2 belong to group B lineage of β-CoV, while MHV and MERS-CoV belong to groups A and C, respectively [12]. The genomes of CoVs contain six to ten open reading frames (ORFs). The first ORF (ORF1a/b) accounts for about two-thirds of the viral genome and mainly encodes replicase proteins [13, 14]. The remaining third of the genome is transcribed into sub-genomic RNAs (sgRNAs), which encode the four structural proteins: spike (S), nucleocapsid (N), membrane (M), and envelope (E) protein. These structural proteins are necessary for the formation of mature virions; M and E proteins also participate in the viral assembly process [15]. As proteinaceous constituents of the helical nucleocapsid, the N proteins bind and encapsulate the genomic RNA resembling a “beads-on-a-string” assembly. The S protein enables the virus to enter host cells.

**Figure 1.**
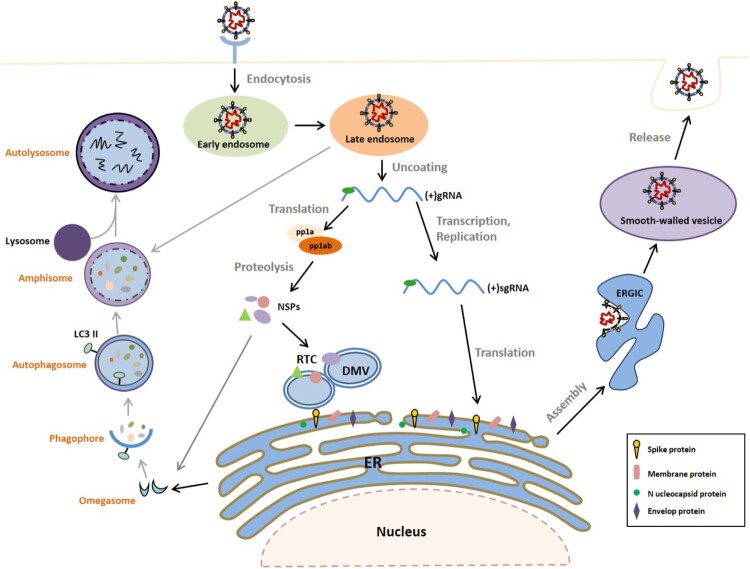
Replication cycle of coronavirus and autophagy pathways. (1) Schematic diagram showing the general replication cycle of CoV. CoV infection begins with the attachment of spike (S) protein to the cognate cellular receptor, which then promotes the fusion of the viral and cell plasma membrane and induces endocytosis. Subsequently, the nucleocapsid is released to the cytoplasm and the gRNA is translated through the ribosome to produce polyproteins pp1a and pp1ab. These are then cleaved by proteases to generate NSPs, which induce the rearrangement of the cellular membrane to form DMVs, to which the viral RTCs are anchored. The viral gRNA is replicated via a negative-sense intermediate, and sgRNA is synthesized by discontinuous transcription. The sgRNAs encode the viral accessory and structural proteins. Particle assembly occurs in the ERGIC, and smooth-walled vesicles bud out to egress via exocytosis. (2) The autophagy pathway. Under various inducing signals (e.g. amino acid starvation and pathogen infection), autophagy is initiated to form isolation membranes (phagophores) through omegasome intermediates. Closure of the isolation membrane results in formation of the autophagosome, which can fuse with late endosome to form an amphisome. Finally, amphisome and lysosome fusion leads to the degradation of autophagosome contents by lysosomal hydrolases in the autolysosome. CoV, coronavirus; EE, early endosome; ERGIC, ER-Golgi intermediate complex; gRNA, genomic RNA; LE /MVB, late endosome /multivesicular body; NSP, non-structural protein; sgRNA, sub-genomic RNA.

After binding of the S protein to the host cell-specific receptor (Figure 1), CoVs enter their host cells by endocytosis followed by release of the genomic RNA into the cytoplasm [13]. The positive-strand RNA immediately serves as a template for ribosomal translation of ORF1a and ORF1b, giving rise to the two poly-protein precursors pp1a and pp1ab, respectively. Subsequently, these poly-proteins are cleaved by viral proteases into 16 non-structural proteins (NSPs), which partially localize to the endoplasmic reticulum (ER) membrane to establish the viral RNA replication-transcription complex (RTC) [12]. CoV synthesizes sgRNAs as well as full-length negative-sense RNA intermediates, which in turn are the templates for the synthesis of progeny virus genomes [16]. The first are translated into the four structural proteins and accessory proteins. Nucleocapsids are formed by the encapsidation of full-length progeny genomes by N proteins [17]. Newly synthesized nucleocapsids are surrounded by the envelope membrane-forming virions. Finally, viral particles are transported to the plasma membrane within smooth-walled vesicles and released from the infected cells by exocytosis [18].

Similar to other positive-strand RNA viruses of eukaryotes, CoV infection hijacks the intracellular membranes of host cells to form double-membrane vesicles (DMVs) [19, 20], which generally reside in the perinuclear region of the cell, creating microenvironments that promote viral RNA synthesis and protect the double-stranded RNA from detection by the innate immune system [21]. Although these autophagosome-like DMVs have been well characterized, the origin of bilayer membrane is still debated, and some studies suggest that autophagy machinery is involved in DMV formation [22, 23]; however, how autophagy is involved in the conversion of host membranes into DMVs is unclear. To a certain extent, the structural similarity between DMVs and autophagosomes prompted the investigation of the interplay between CoV infections and autophagy.

## Autophagy

Macroautophagy (also known as autophagy) is a self-degradation process that is conserved among eukaryotic cells. Under starvation conditions or when damaged organelles, invaded pathogens, and misfolded or long-lived proteins accumulate in cells, autophagy is activated through a signalling process that effectively degrades these substrates and provides nutrients, such as amino acids, through recycling. Therefore, autophagy plays a vital role in maintaining intracellular homeostasis [24, 25]. During the autophagic process, substrates like damaged organelles in the cytoplasm are first surrounded by double-layered membrane structures named autophagosomes that can fuse with the late endosomes or lysosomes. Finally, the content within the autophagosomes is degraded by the proteolytic enzymes in the lysosomes [25].

As a part of the cell’s defence system, autophagy protects against bacterial or viral infection by delivering them for lysosomal degradation. Autophagy also plays a pivotal role in pathogenic antigen presentation, relative cytokine secretion, and T/B cell differentiation for activating innate or adaptive immune responses [26].

**Figure 2.**
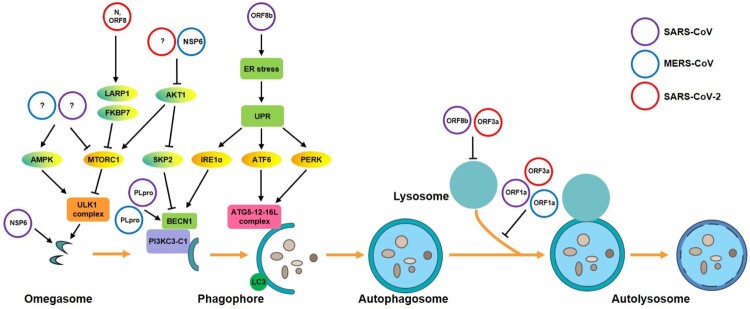
A proposed model of the interaction of emerging human coronaviruses and autophagy in host cells. Emerging human CoVs (SARS-CoV, MERS-CoV, or SARS-CoV-2) modulate multiple phases of autophagy in infected host cells. (1) At the start of the autophagic process, SARS-CoV and SARS-CoV-2 NSP6 proteins induce the formation of omegasome intermediates. Moreover, all three CoVs can activate the ULK1 complex to promote phagophore formation via the AMPK/MTOR signalling pathway. (2) MERS-CoV and SARS-CoV-2 facilitate AKT1/SKP2-dependent degradation of BECN1 to inhibit vesicle nucleation. Conversely, SARS-CoV and MERS-CoV PLpro deubiquitinates BECN1 to promote autophagy induction. (3) SARS-CoV NSPs and spike (S) proteins cause ER stress, triggering the UPR, and then promote phagophore elongation by activating ATG5-12-16L complex. (4) SARS-CoV and MERS-CoV PLpro induces incomplete autophagy by impairing autophagosome maturation by blocking the fusion of autophagosomes and lysosomes. Additionally, SARS-CoV ORF8b can cause lysosomal damage. UPR, unfolded protein response; ORF, open reading frame; PI3KC3-C1, class III phosphatidylinositol 3-kinase complex I; PLpro, papain protease.

Depending on the type of virus and the corresponding host cell, autophagy activated during viral infections can either benefit or impede virus replication. Some viruses, such as human cytomegalovirus, coxsackievirus B3, and herpes simplex virus type 1, inhibit autophagy [27–29]. Conversely, other viruses, such as hepatitis B virus, human immunodeficiency virus, dengue virus, and influenza virus, facilitate their replication and maturation by enhancing the autophagy [30–34]. The interaction between different CoV infections and autophagy has recently attracted a lot of attention. Collectively, different reports have given rise to two major concerns: if and how autophagy is affected by CoV infection, and whether autophagy is involved in the replication of CoVs. In the following sections, we summarize and discuss the latest research substantiating the interplay between autophagy and each of the three emerging hCoVs (Figure 2) as well as MHV.

## Coronavirus infections and autophagy

### MHV infection and autophagy

Unlike the strict limitations for studying the three emerging hCoVs, the model virus MHV is widely used in many laboratories worldwide. Some previous studies have shown that autophagy is induced through different mechanisms during MHV infection (Table 1) and ,in turn, plays a crucial role in the viral lifecycle. Cottam et al. reported that MHV NSP6 can activate autophagic flux to generate autophagosomes from the ER through omegasome intermediates, and MHV is unable to generate DMVs in mouse embryonic stem cells lacking the autophagy-related gene *ATG5* [7]. Another study by Prentice et al. revealed that MHV replication is decreased more than 1000-fold in *ATG5*^−/−^ embryonic stem cells compared to those expressing ATG5, which suggests that autophagy greatly influences DMV formation and MHV replication [35]. In contrast to the study in embryonic stem cells, Zhao et al. found that MHV-induced membrane rearrangements do not require the participation of either ATG5 or the conversion of autophagy component LC3-I to LC3-II, and the intact autophagic pathway is not essential for viral replication in primary murine embryonic fibroblasts (pMEFs) [36]. Moreover, a subsequent important study from Reggiori et al. also demonstrated that MHV may utilize LC3-I in a non-canonical manner [22]. The outer membrane of DMVs contains large amounts of LC3-I, which acts as a coat protein, whereas autophagosomes also contain LC3-II as a marker protein [22, 37]. Thus, a novel role for LC3-I was further unveiled in the formation of both ER-associated degradation (ERAD) tuning vesicles (EDEMosomes) and MHV-induced DMVs, in an autophagy-independent manner [22]. It is important to highlight that these different studies have been carried out in different MHV-infected cell lines, indicating that the relationship between autophagy and MHV infection may be cell-specific. Collectively, MHV infection induces autophagy formation; however, MHV infection and replication may not require autophagy function but may involve some autophagy-related proteins and ATGs in a non-canonical autophagy manner.

**Table 1.** Effects of emerging human coronavirus infections on autophagy.

CoV	Proteins	Models	Impacted sites	Results	References
MHV, SARS-CoV	NSP6	Vero cells	MTOR kinase and autophagosomes	Limit expansion of autophagosomes	[6]
MHV, SARS-CoV	NSP6	Vero /HEK293 cells	ER membrane	Generate autophagosomes from the ER via an omegasome intermediate	[7]
MHV	?	Mouse embryonic fibroblasts	ER membrane	Utilize LC3-I-positive EDEMosomes to form DMVs that provide the membranous scaffold for the rirus replication	[22]
MHV	RTC	Murine embryonic stem cell and delayed brain tumour cells	ATG5	Activate autophagy and form DMVs	[35]
SARS-CoV, MERS-CoV	PLpro	HEK293T cells	BECN1 activity and the fusion of autophagosomes and lysosomes	Protect BECN1 and reduce the degradation of autophagy	[40, 43]
SARS-CoV	ORF8b	Vero /HeLa cells	TEEB and lysosomal stress	Enhance autophagic flus and caused lysosomal damage	[41]
SARS-CoV	?	ACE2 knockout mice	AMPK/MTOR signalling pathways	Induce autophagy formation	[75, 76]
SARS-CoV	Spike protein	Vero cells	ER membrane	Trigger UPR and induce autophagy	[77]
MERS-CoV	NSP6	HEK293 /Vero B4 cells	BECN1 activity and the fusion of autophagosomes and lysosomes	Inhibit autophagy and block autophagosome-lysosome fusion	[8]
SARS-CoV-2	N and ORF8	HEK293T cells	PI3KC3 complex	Initiate autophagy	[49]
SARS-CoV-2	?	NCI-H1299 /Vero FM cells	AMPK/MTOR and AKT1/SKP2 signalling pathways	Inhibit autophagy	[50]
SARS-CoV-2	ORF3a	HEK293T /HeLa cells	HOPS component VPS39 or autophagy regulator UVRAG; Lysosome damage	Block autolysosome formation; Impair lysosomal function.	[51, 52]

### SARS-CoV infection and autophagy

Existing evidence supports that SARS-CoV infection can increase autophagosome formation in host cells (Table 1). SARS-CoV infection may induce autophagy by specifically down-regulating the expression of the viral entry receptor ACE2 [39–41]. It is assumed that SARS-CoV infection leads to AMP-protein activated kinase (AMPK) activation or MTOR inhibition to activate autophagic flux by reducing the expression of ACE2 [40], which is an important member of the renin-angiotensin system and regulates multiple physiological activities in the body. Several SARS-CoV proteins have been shown, *in vitro,* to cause autophagy induction via various mechanisms. Cottam et al. found that SARS NSP6, a multiple membrane-spanning protein that is located in the ER, partially co-localized with LC3-positive structures, suggesting that it may be released from the ER into autophagosomes [7]. They further demonstrated that NSP6 activated autophagy by increasing phosphatidylinositol 3-phosphate (PtdIns3P) levels, and recruited the effector protein double-FYVE-containing protein 1 (DFCP1), which caused the generation of autophagosomes from the ER through an omegasome intermediate. Cottam et al. also observed that NSP6 induced higher numbers of autophagosomes per cell compared to processes activated by starvation, but the expansion of generated autophagosomes was obviously reduced compared with normal ones [6]. Additionally, the papain-like protease (PLpro) encoded by SARS-CoV ORF1a can interact with and deubiquitinate the autophagy regulator BECN1 to protect it from degradation, thereby prolonging its stability [38–40]. Notably, the intracellular aggregation of SARS-CoV ORF8b triggers cellular stress by activating the transcription factor EB and its target genes, which also leads to enhanced autophagic flux [41, 42]. However, large amounts of ORF8b may also cause lysosomal damage, destroy autolysosomal homeostasis, and reduce the ability of the cell to degrade cargo proteins. Therefore, the aggregation propensity of viral ORF8b may be a strategy to protect SARS-CoV from degradation. Moreover, Chen et al. also demonstrated that the transmembrane-associated papain-like protease (PLpro-TM) induces incomplete autophagy by impairing autophagosome maturation and blocking the fusion between autophagosomes and lysosomes, resulting in the intracellular accumulation of a large number of autophagosomes [43]. Collectively, due to the fact that individual SARS-CoV proteins cause autophagy induction or disturb autophagosome maturation, SARS-CoV infection is a potent activator of autophagosome formation and accumulation.

While SARS-CoV infection can increase autophagosome formation, whether autophagy is necessary for viral replication is still under debate. Similar to MHV infection, the formation of DMVs was observed in SARS-CoV-infected HEK293T-ACE2 cells [44]. Although SARS-CoV-induced DMVs and NSPs co-localize with autophagy marker LC3 [45], LC3-I is believe to play an autophagy-independent function in the formation of both EDEMosomes and DMVs. Instead, Snijder et al. failed to detect the co-localization of LC3 or GFP-LC3 with RTCs in SARS-CoV-infected Vero cells [46]. Similar to the transmembrane-associated papain-like protease 2 (PLP2-TM) of porcine epidemic diarrhoea virus (PEDV), PLpro-TM of SARS-CoV or MERS-CoV may also directly interact with BECN1 to sequester the critical innate immune signalling adapter STING to the autophagosomes, which would impede downstream antiviral responses and thereby promote viral replication [43]. Thus, this study supports the notion that SARS-CoV may utilize BECN1-induced autophagy for viral immune escape. Due to the limited evidence available, the necessary requirement of autophagy in SARS-CoV infection and replication remains elusive and further studies are needed.

### MERS-CoV infection and autophagy

To date, several lines of evidence indicate that the MERS-CoV infection is related to disturbed autophagy (Table 1). Firstly, MERS-CoV infection was shown to block autophagosome-lysosome fusion by interfering with the interactions of SNARE complex proteins STX17-VAMP8-SNAP29, mediated mainly by NSP6. The PLpro-TM of MERS-CoV, which associates with LC3 and BECN1, was also reported to induce incomplete autophagy and cause the accumulation of autophagosomes by impairing the process of autophagosome-lysosome fusion in different cell lines [43]. Additionally, Gassen et al. demonstrated that MERS-CoV decreased cellular BECN1 levels, but increased K48-polyubiquitinylation of BECN1 through the induction of phosphorylation of E3 ubiquitin ligase S-phase kinase-associated protein 2 (SKP2) in MERS-CoV-infected Vero B4 cells [8]. However, similar to its SARS-CoV NSP3 counterpart, MERS-CoV NSP3 possesses a PLpro domain that interacts with and deubiquitinates BECN1 to increase its abundance [38–40], which is beneficial for autophagy initiation. Collectively, existing evidence mainly supports that MERS-CoV infection impairs autophagy formation and maturation via several viral proteins in different ways.

Unfortunately, the exact role of autophagy in MERS-CoV infection is still unclear. Maejima et al. demonstrated that deletion of the *ATG5* gene in Vero B4 cells facilitated MERS-CoV replication 6-fold and promoted infectious viral particle formation 52-fold [8]. They further found that SKP2 inhibition by small specific molecule inhibitors not only increased BECN1 stability and enhanced autophagy but also reduced the replication of MERS-CoV up to 28,000-fold. Consequently, the therapeutic induction of autophagy via SKP2 inhibition has emerged as a promising treatment strategy. In addition, Kindrachuk et al. reported that MERS-CoV replication is significantly reduced *in vitro* upon treatment with kinase inhibitors targeting the ERK/MAPK and PI3K/AKT/MTOR signalling pathways [47]. Hence, we speculate that viral replication in MERS-CoV-infected cells is significantly decreased by autophagy induction by the PI3K/AKT/MTOR kinase inhibitors. Collectively, enhanced autophagy can reduce the replication of MERS-CoV, indicating that autophagy may be a new therapeutic target for controlling MERS-CoV infection.

### SARS-CoV-2 infection and autophagy

As a new emerging hCoV, it is largely unknown how SARS-CoV-2 is targeted by or manipulates autophagy. Accumulating evidence suggests that SARS-CoV-2 infection may perturb the autophagic process (Table 1). Bouhaddou et al. revealed through a quantitative mass spectrometry-based phosphoproteomics survey in SARS-CoV-2-infected Vero E6 cells that the p38/MAPK signalling pathway was activated, while PIK3CA/AKT was inhibited, both of which are bound to autophagy induction [48]. Another recent study by Gordon et al. identified host factors related to autophagy, by mapping the SARS-CoV-2 interactome in human host cells using affinity-purification coupled to protein identification by mass spectrometry [49]. They further showed that the N and ORF8 proteins of SARS-CoV-2 lead to MTORC1 inhibition by separately interacting with the MTORC1 pathway proteins La ribonucleoprotein 1, translational regulator (LARP1), and FKBP prolyl isomerase 7 (FKBP7), but not with the MTORC1 complex itself [49]. Viral N and ORF8 could stimulate autophagy initiation; however, this needs further verification in future studies. However, a preprint study from Gassen et al. reported that SARS-CoV-2 infection restricts autophagy formation by interfering with multiple metabolic pathways *in vitro* [50]. They further demonstrated that SARS-CoV-2 reduces glycolysis and protein translation by limiting the activation of AMPK/MTORC1 signalling. SARS-CoV-2 infection also decreases autophagy-inducing spermidine and facilitates the AKT1/SKP2-dependent degradation of BECN1. Importantly, two recent studies from different laboratories reported that SARS-CoV-2 infection increased autophagosome formation, but impaired autophagosome maturation [51, 52]. Subsequently, they further revealed that ORF3a of SARS-CoV-2 alone was sufficient to trigger an incomplete autophagy response by blocking the fusion of autophagosomes and lysosomes. Qu et al. found that SARS-CoV-2 ORF3a directly interacts with the autophagy regulator UVRAG to selectively inhibit the formation of the BECN1-VPS34-UVRAG complex [52], while Miao et al. found that ORF3a directly interacts with the HOPS component VPS39 to block the interaction of HOPS with autophagosomal STX17 as well as destroy lysosomal function [51]. Interestingly, both of these two studies found that SARS-CoV ORF3a failed to increase autophagosome formation and impair autophagosome maturation, indicating that ORF3a-induced incomplete autophagy is a unique signature of SARS-CoV-2 infection. Altogether, the evidence so far suggests that the SARS-CoV-2 infection is related to the induction of incomplete autophagy. Nevertheless, the specific mechanisms by which SARS-CoV-2 modulates autophagy require further investigation.

To date, there is limited evidence that the autophagy machinery modulates the infection or replication of SARS-CoV-2. Several studies using autophagy-related drugs/compounds suggest that activating autophagy exerts protective effects against SARS-CoV-2 infection and replication. For instance, Bouhaddou et al. recently reported that the pharmacological inhibitors of p38/MAPK signalling and PIKFYVE downstream of PIK3CA/AKT were found to possess strong antiviral efficacy in Vero E6 and A549-ACE2 cells infected with SARS-CoV-2 [48]. Rayner et al. also demonstrated that AR12 (OSU-03012), a derivative of celecoxib, suppressed the production of infectious virions by enhancing autophagic flux in SARS-CoV-2 infected Vero cells [53]. Additionally, a recent preprint article from Gassen et al. presented that autophagy activation can suppress viral propagation in SARS-CoV-2 infected NCI-H1299 and Vero FM cells by using the pro-autophagic compound spermidine and the AKT inhibitor MK-2206 [50]. Since these drugs targeting autophagy induction, autophagy activation could exert protective effects on SARS-CoV-2 infection and replication. However, a recent preprint study from Qu et al*.* reported that a significant reduction in viral yield was observed in SARS-CoV-2 infected *ATG3* and *ATG5*-deficient MEF-ACE2 cells, indicating that cell-specific autophagic responses may exist. Indeed, this preliminary report has not been peer-reviewed and the effect of deficient essential autophagy genes was not further investigated in other SARS-CoV-2 infected cell lines. Additionally, three independent preprint studies based on full-genome loss of function CRISPR screens recently demonstrated that SARS-CoV-2 can hijack autophagy-related genes transmembrane protein 41B (TMEM41B) or 106B (TMEM106B) to promote viral infection and replication, while the intact autophagy pathway is not required [54–56]. These works suggest that some relevant autophagy-related genes are essential for SARS-CoV-2 infection and replication in an autophagy-independent manner. Taken together, the available evidence suggests a relevant role of autophagy in SARS-CoV-2 infection and replication, but further studies are need to identify its effect on SARS-CoV-2 infection/ replication and elucidate its mechanisms.

## Targeting autophagy as a promising strategy for COVID-19

As there are no approved specific antiviral drugs or preventative vaccines for COVID-19 currently available, prevention and control strategies to limit the spread of the COVID-19 epidemic are critical. Scientists have started trials on potential prophylactic and treatment options [57, 58]. Among these strategies, the repurposing of approved or candidate drugs, including autophagy based-drugs, might represent a shortcut to develop treatments against SARS-CoV-2.

To date, the development of novel therapeutic strategies for COVID-19 has achieved great progress. Some autophagy based-drugs derived from FDA-approved drugs, compounds in clinical trials, or preclinical compounds were shown to be effective against SARS-CoV-2 *in vitro* [53]. Firstly, AR12 exhibited potent antiviral activity in SARS-CoV-2-infected Vero cells by increasing autophagic flux and enhancing virus protein degradation, which was associated with degradation of chaperone GRP78 [53]. This supported the re-entry of AR12 into the clinical therapeutic regimen of SARS-CoV-2 infection. Admittedly, except for inducing autophagy, AR12 has been shown to be effective in suppressing cell proliferation and inducing apoptotic death by decreasing epidermal growth factor receptor (EGFR) expression or preventing Y-box binding protein-1 (YB-1) from binding to the *EGFR* promoter at the 1b and 2a sites [59, 60]. Moreover, the pharmacological inhibitors of p38/MAPK signalling, including gilteritinib, ralimetinib, MAPK13-IN-1, and ARRY-797, as well as apilimod, a specific inhibitor of PIKFYVE downstream of PIK3CA/AKT activity, were found to possess strong antiviral efficacy in SARS-CoV-2-infected Vero E6 and A549-ACE2 cells [48], indicating that autophagy induction by the inhibition of p38/MAPK and PIK3CA/AKT signalling could control viral replication. Besides inhibition of SARS-CoV-2 replication, inhibition of p38/MAPK signalling may also suppress harmful pro-inflammatory cytokine production caused by viral infection, which is related to SARS-CoV-2 pathogenesis [61]. Additionally, a preprint study reported that exogenous administration of autophagy inducer spermidine, AKT inhibitor MK-2206, and the Beclin-1 stabilizing drug niclosamide suppressed SARS-CoV-2 replication through autophagy activation in SARS-CoV-2-infected Vero FM cells [50]. These three autophagy related drugs may also have other physiological functions. For instance, both spermidine and niclosamide are potent to neutralize endosomal/lysosomal acidification, which may contribute to prevent virus entry into host cells [62, 63]. Particularly, these clinically approved and well-tolerated autophagy-related drugs do give us hope of potential clinical evaluation for combating SARS-CoV-2 infection. However, considering these aforementioned drugs may have various physiological functions, possible pleiotropy should be investigated for all pharmacological candidates against SARS-CoV-2 viral infection.

Notably, antimalarial drugs chloroquine (CQ) and hydroxychloroquine (HCQ), as classical autophagy inhibitors that impair autophagosome maturation, exerted strong anti-SARS-CoV-2 effects in SARS-CoV-2 infected Vero E6 cells [64, 65]. Therefore, they were highly recommended to be repurposed for the treatment of COVID-19 this past year. Unfortunately, their inhibitory efficiency did not extend to SARS-CoV-2 infected TMPRSS2-expressing human lung cells or animal models and COVID-19 patients [66–69]. It should be noted that lysosomotropic agents CQ and HCQ have many physiological functions, including blocking autophagosome-lysosome fusion, alkalizing endosomal/lysosomal acidification, and disrupting lipid clustering and ACE2 localization [70, 71]. It is doubtful that their putative effects on autophagy inhibition are necessarily causal for their anti-SARS-CoV-2 activity in specific cells. To date, the definite mechanism of their action against SARS-CoV-2 infection/replication in specific cells has not been elucidated yet.

Collectively, existing evidence indicates that the modulation of autophagic processes by using autophagy-related drugs/compounds could be an effective approach in inhibiting SARS-CoV-2 infection/replication, but further studies are urgently needed.

## Conclusions and perspectives

In the twenty-first century, public health emergencies of international concern caused by three emerging hCoV infections have not only brought inestimable economic loss globally but also seriously undermined social stability worldwide [72]. To control the spread and recurrence of the COVID-19 epidemic, effective therapeutic drugs and preventive vaccines are urgently needed. In addition, before a new emerging viral disease occurs, it is essential to develop effective broadly acting antiviral therapies, such as drugs targeting the autophagy process [65, 73].

In this review, we examined the current evidence suggesting that emerging hCoV infections affect several steps of autophagy [65, 74]. Conversely, autophagy may have an inhibitory effect on viral infection or replication. Nevertheless, the exact mechanisms underlying the role of autophagy in the emerging hCoV life cycle need further investigation. Some autophagy-based inhibitors demonstrate encouraging therapeutic potential against SARS-CoV-2 infections [50, 53]. Therefore, repurposing of approved and well-tolerated drugs targeting autophagy would offer a welcome shortcut to rapidly develop treatments against COVID-19. Collectively, autophagy processes may be targeted to combat SARS-CoV-2 infections and may become an important component of drug combination therapies to improve the treatment outcomes for COVID-19.
